# Influenza Virus in Human Exhaled Breath: An Observational Study

**DOI:** 10.1371/journal.pone.0002691

**Published:** 2008-07-16

**Authors:** Patricia Fabian, James J. McDevitt, Wesley H. DeHaan, Rita O. P. Fung, Benjamin J. Cowling, Kwok Hung Chan, Gabriel M. Leung, Donald K. Milton

**Affiliations:** 1 Work Environment Department, University of Massachusetts Lowell, Lowell, Massachusetts, United States of America; 2 Department of Environmental Health, Harvard School of Public Health, Boston, Massachusetts, United States of America; 3 Pulmatrix Inc., Lexington, Massachusetts, United States of America; 4 Department of Community Medicine and School of Public Health, Li Ka Shing Faculty of Medicine, The University of Hong Kong, Hong Kong, China; 5 Department of Microbiology, The University of Hong Kong and Queen Mary Hospital, Hong Kong, China; Erasmus Medical Center, Netherlands

## Abstract

**Background:**

Recent studies suggest that humans exhale fine particles during tidal breathing but little is known of their composition, particularly during infection.

**Methodology/Principal Findings:**

We conducted a study of influenza infected patients to characterize influenza virus and particle concentrations in their exhaled breath. Patients presenting with influenza-like-illness, confirmed influenza A or B virus by rapid test, and onset within 3 days were recruited at three clinics in Hong Kong, China. We collected exhaled breath from each subject onto Teflon filters and measured exhaled particle concentrations using an optical particle counter. Filters were analyzed for influenza A and B viruses by quantitative polymerase chain reaction (qPCR). Twelve out of thirteen rapid test positive patients provided exhaled breath filter samples (7 subjects infected with influenza B virus and 5 subjects infected with influenza A virus). We detected influenza virus RNA in the exhaled breath of 4 (33%) subjects–three (60%) of the five patients infected with influenza A virus and one (14%) of the seven infected with influenza B virus. Exhaled influenza virus RNA generation rates ranged from <3.2 to 20 influenza virus RNA particles per minute. Over 87% of particles exhaled were under 1 µm in diameter.

**Conclusions:**

These findings regarding influenza virus RNA suggest that influenza virus may be contained in fine particles generated during tidal breathing, and add to the body of literature suggesting that fine particle aerosols may play a role in influenza transmission.

## Introduction

Although the pathogen responsible for human influenza virus infection was described over 70 years ago, “our understanding of the transmission of influenza” has recently been characterized as “woefully inadequate” [1]. The US Department of Health and Human Services Pandemic Influenza Preparedness Plan [2] concluded that the relative importance for influenza transmission of direct contact, large droplet, and airborne small particles is not known. Two recent reviews in respected journals reached opposite conclusions about how influenza is transmitted [3], [4]. After reviewing the same epidemiology, animal model, and case study data, one review classified influenza as an opportunistically, and only rarely, airborne transmitted disease while the other review suggested that influenza is preferentially or obligatorily airborne transmitted[5]. An Institute of Medicine report in 2007 stated that the “paucity of definitive data on influenza transmission is a critical gap in the knowledge base needed to develop and implement effective prevention strategies”[1].

Little is known about the aerosols produced by influenza-infected subjects. In studies of exhaled breath particles from healthy subjects during tidal breathing, researchers reported concentrations from 1 to >10,000 particles per liter, with the majority less than 0.3 µm in diameter [6]–[8]. One of these studies also reported that 55% of the population studied exhaled >98% of the particles and concluded that these subjects–classified as “high producers”–could over time exhale more particles during normal tidal breathing than during relatively infrequent coughing or sneezing events [8]. If particles exhaled during tidal breathing contain infectious viruses, Edwards' finding may have important implications for airborne transmission of infectious diseases. In animal models, influenza infection by airborne transmission has been demonstrated in monkeys [9], ferrets [10]–[12], mice [13]–[20], and guinea pigs [21], [22]. Recent work by Huynh et al showed recovery of rhinovirus and parainfluenza virus via PCR from infected patients who coughed and breathed through masks made with electret [23]. Although they also studied subjects infected with influenza virus, none was recovered.

To address gaps in our knowledge regarding the generation of influenza virus aerosols, we report the concentration of influenza virus RNA in the exhaled breath of persons infected with influenza, and characterize their exhaled breath particle production during tidal breathing.

## Results

A total of 68 rapid flu tests were administered from July 23 through September 14, 2007: thirty-six were collected at Site A, thirty at site B, and two from Site C. Over half (55%) of the screened population was male, the average age was 35 years (SD = 12), the average body temperature was 38.1 (SD = 0.7). Only 9% of the screened subjects had been vaccinated for the 2007 season, and 8% reported having at least one influenza vaccine in a previous influenza season. Thirty six of 68 subjects given the rapid test completed the symptoms part of the questionnaire and of these over 80% reported having cough, headaches, fatigue, and sore throat.

### Exhaled breath results

Thirteen (19%) of the subjects tested positive for influenza using the rapid test and were asked to participate in the exhaled breath study. According to the rapid test and confirmatory PCR, 5 subjects were infected with influenza A virus and 7 subjects were infected with influenza B virus. Twelve of the 13 subjects provided exhaled breath filter samples; one subject reported feeling too fatigued and did not participate. Field blank filters were collected from two uninfected individuals. Analysis of the flow-time record supported the observation by field technicians that none of the subjects coughed during filter collection.

We detected influenza virus RNA in the exhaled breath of 4 (33%) subjects: three (60%) of the five patients infected with influenza A virus and one (14%) of the seven infected with influenza B virus. There was no correlation between nasal and throat swab influenza virus RNA concentrations and exhaled breath influenza virus RNA concentrations. Table 1 presents the demographic characteristics and symptoms of subjects, stratified according to whether they had detectable influenza virus RNA in their exhaled breath.

**Table 1:** Demographic characteristics and symptoms of subjects with and without detectable influenza virus RNA in exhaled breath.

	Exhaled breath negative	Exhaled breath positive
Total number	8	4
Male	75%	75%
Average age (years) (SD*)	37 (14)	34 (19)
Age range (years)	19–61	14–51
Body temperature (°C) (SD*)	38.3 (0.4)	38.7 (0.6)
Symptom onset		
1–2 days past	75%	50%
3–5 days past	25%	50%
Vaccinated this season	13%	0%
*Symptoms*		
Cough	100%	75%
Sputum changes**	13%	25%
Fatigue	100%	100%
Malaise	63%	75%
Headache	100%	100%
Sore throat	100%	75%
Diarrhea	0%	0%
Dyspnea	25%	25%
Chills	63%	50%
Sweats	63%	50%

* SD = standard deviation

** Increase in production or changes in character (color, consistency)

Concentrations in exhaled breath samples ranged from <48 to 300 influenza virus RNA copies per filter on the positive samples, corresponding to exhaled breath generation rates ranging from <3.2 to 20 influenza virus RNA copies per minute. Table 2 presents the influenza virus type, viral RNA exhalation rate, and viral RNA copies detected per well in each qPCR replicate from the exhaled breath analysis of 12 tested subjects. All influenza A virus samples were hemagglutinin type H3. Laboratory blanks and the two field blanks collected were negative.

**Table 2:** Influenza virus type, results for each qPCR replicate, and exhalation rate.

		qPCR of Filter Extract^a^			
Subject ID	Influenza virus type (sub-type)	Replicate 1	Replicate 2	Replicate 3	Influenza virus RNA exhalation rate^b^
A-06	A (H3)	47	21	44	20
A-07	A (H3)	ND	ND	<6	<3.2
A-08	B	ND	ND	ND	ND
A-11	B	ND	ND	ND	ND
A-21	A (H3)	ND	ND	ND	ND
A-23	A (H3)	ND	ND	<6	<3.2
A-24	B	ND	7	ND	<3.2
A-25	B	ND	ND	ND	ND
A-34	B	ND	ND	ND	ND
B-01	A (H3)	ND	ND	ND	ND
B-09	B	ND	ND	ND	ND
B-25	B	ND	ND	ND	ND
A-37 (control)		ND	ND	ND	ND
A-38 (control)		ND	ND	ND	ND

a Number of influenza RNA copies detected per well (5 µl cDNA per well).

b Influenza virus RNA copies/ minute

ND = not detected by qPCR; limit of quantification was 6 influenza virus RNA copies per qPCR well when all three replicates were detected.

### Particle counts

**Figure 1:**
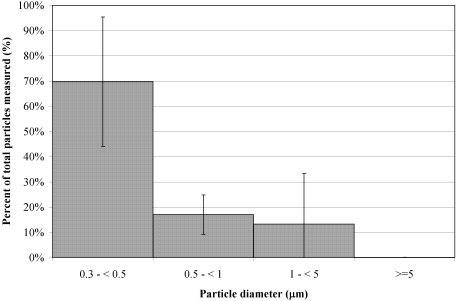
Exhaled breath particle size distribution averaged from 10 influenza infected subjects.

Exhaled breath particle size and number data was obtained for 10 of the 12 subjects who provided filter samples. Data from the two remaining subjects, including the subject with the highest concentration of exhaled influenza virus RNA, could not be analyzed because of mask leaks. Across all subjects, total particle concentrations ranged from 67 to 8,500 particles per liter of air. Particle concentrations in the size selective bins ranged from 61 to 3,848 L^−1^ (particles between 0.3 µm and <0.5 µm), 5 to 2,756 L^−1^ (0.5 µm and <1 µm), 1 to 1,916 L^−1^ (1 µm and <5 µm), and 0 to 9 L^−1^ (≥5 µm). Figure 1 presents the averaged size distribution of the particles measured from all 10 subjects. On average 70% of the particles measured were between 0.3 µm and <0.5 µm, 17% between 0.5 µm and <1 µm, and 13% between 1 µm and <5 µm. Particles larger than 5 µm were rarely recorded (<0.1%). Of the 10 subjects, 50% exhaled more than 500 particles per liter of air. Two control samples were collected from asymptomatic subjects who were not infected with influenza. Both controls exhaled more than 500 particles per liter of air.

## Discussion

We detected influenza virus RNA in the exhaled breath of 33% of subjects with laboratory-confirmed influenza. Few previous studies have examined microorganisms in exhaled breath: Couch et al. published two reports of finding infectious Coxsackievirus in large droplets and droplet nuclei generated by coughs and sneezes [24]–[26]; Fennelly et al. recovered *Mycobacterium tuberculosis* from infected patients who coughed into a collection box [27]; Downie et al recovered infectious variola virus from smallpox patients who talked next to an impinger [28]; Duguid et al. recovered indicator oral bacteria from healthy subjects during nasal breathing, talking, coughing, and sneezing, but none were recovered during mouth breathing [29]. More recently Huynh et al looked for rhinovirus, parainfluenza and influenza viruses from infected subjects who breathed, talked and coughed through electret [23]. Via PCR they were able to recover rhinovirus RNA and parainfluenza virus RNA from coughing and talking maneuvers, and rhinovirus RNA from tidal breaths; no influenza virus RNA was recovered. To the best of our knowledge, previous exhaled breath studies have not reported detection of influenza virus RNA aerosols during tidal breathing.

Rudnick and Milton [30] used data from the influenza outbreak aboard an aircraft reported by Moser et al [31] and the Wells-Riley equation for non-steady-state conditions [32] to estimate a quantum generation rate of from 79 to 128 quanta per hour for a highly infectious influenza case. In this equation a quantum is the infectious dose needed to initiate disease in 63% of exposed subjects, is equivalent to an ID_63_, and can contain a number of infectious viruses [33]. In the present study, we measured generation rates of <192 and 1200 influenza virus RNA copies per hour for subjects with detectable influenza virus RNA in their breath. Assuming each RNA copy represents one infectious virus, and if, as Alford et al suggested [34], [35], the human infectious dose 50% (ID_50_) by aerosol is between 0.6 and 3 TCID_50_, then one quanta equals approximately one TCID_50_. This implies that the quantum generation rate we measured in our subjects is 10-fold higher than the Rudnick and Milton estimate for a “superspreader”. However, not all viral RNA copies detected by PCR are necessarily infectious. We found that our laboratory virus stocks have a ratio of 300 virus particles per infectious virus (characterized using real-time PCR and a cell culture-based infectivity assay, data not shown)–a ratio consistent with reports from other laboratories [36]–[38]. Thus the generation rate of infectious viruses may be as little as 1/300^th^ of the total number determined by PCR and the resulting estimates of quantum generation rate (<0.64 to 4 quanta/h) from our data are lower than those estimated for a superspreader by Rudnick and Milton. However, estimates based on tissue culture virus to RNA ratios may be overly conservative because clinical specimens and aerosols may have fewer defective viruses and less viral nucleic acid which is not associated with virus particles.

Possible explanations for not detecting influenza virus RNA in a larger proportion of subjects may be due to short sample collection times, the large heterogeneity in the virus production among infected patients (between 10^2^ and 10^7^ TCID_50_/ml of nasopharyngeal fluid on the 2^nd^ day following infection [39]), and the detection limit for our qPCR method. The qPCR method can quantify as few as 6 influenza virus RNA copies per qPCR well, corresponding to 48 influenza virus RNA copies per filter or an exhaled breath generation rate of 3.2 influenza virus RNA copies per minute for a 15 minute sample. For the PCR to be considered quantifiable in our study, all three sample replicates had to cross the fluorescence signal threshold. Samples for which only one or two of the replicates crossed the threshold were considered positive but not quantifiable, and labeled <3.2 influenza virus RNA copies/min (Table 2). With this criterion only one subject had a quantifiable generation rate of 20 influenza virus RNA copies/min, three generated less than 3.2 influenza virus RNA copies/min and the remaining either didn't exhale influenza virus aerosols, or generated undetectable numbers of influenza virus RNA copies/min.

Influenza-infected subjects exhaled from 67 to 8,500 particles per liter of air (geometric mean = 724); 50% of subjects exhaled more than 500 particles per liter, a suggested threshold for identification of high particle producers [8]. These results are consistent with a previous study that measured particle concentrations of 14 to 3,230 particles per liter and also reported 54% high particle producers among healthy adults using similar optical counting methods [8]. Other studies of exhaled breath reported particle concentrations of 20 and 400 particles per liter [40], or a geometric mean of 230 particles per liter [6]. The numbers of subjects in the published exhaled breath studies to date have all been less than eleven. Larger studies of exhaled breath particle concentrations are needed to better characterize the population distribution of particle generation rates. Our study also had a small number of subjects, and we do not have enough information to suggest a relationship between particle production and the probability of influenza virus RNA detection.

Although we don't know whether the RNA we detected originated from free nucleic acid, infectious, or non-infectious viruses, the data presented here show that aerosols of influenza virus origin are generated during tidal breathing. Our sampling protocol required subjects to tidal breathe and patients did not cough during sample collection. The data also suggest that influenza virus RNA is contained in fine particles because over 87% of the exhaled particles were under 1 µm and less than 0.1% were larger than 5 µm. This distribution of particle sizes is consistent with previous studies showing that 98% of particles produced during normal breathing are under 1 µm [6]–[8]. Thus, based on the particle size distribution, it is unlikely that the viral RNA detected was carried on large particles. We also note that the Exhalair sampler fitted with a face mask as the patient interface would likely impact most large particles (>20 µm) on the mask or in tubing connecting the mask to the particle counter and filter cassette. Therefore, it is likely that the influenza virus RNA we detected in exhaled breath was contained in exhaled breath particles <5.0 µm in diameter. If influenza virus is carried in the smaller particles and transmission occurs via the airborne route, the use of interventions such as surgical-type masks as personal protective equipment may not prevent transmission. However, larger studies should be conducted to provide baseline data on particle and infectious influenza virus generation, as well as potential changes in virus generation due to use of surgical masks by patients or healthcare professionals.

In order to study infectious viruses we need an efficient viral aerosol sampler. Most bioaerosol samplers are not suitable for recovering infectious viruses from exhaled breath due to short period collection times, low flow rates, or collection substrates which adversely affect virus infectivity. Other sampler limitations include losses due to particle bounce, as is the case with many impaction type particle collectors, and loss of collection media over time, as is the case with liquid impingers. A new sampler should combine efficient capture of small particles with liquid collection media. Larger similar studies to the one reported in this manuscript are needed to obtain information on particle generation, infectious and non-infectious virus concentrations in exhaled breath in order to estimate influenza virus quantum generation rates, generation variability, and relationship to host and virus characteristics.

### Conclusions

The relative importance of various modes of influenza transmission continue to be debated [3], [4]. We detected influenza virus RNA in the exhaled breath of 4 out of 12 influenza patients and found that >99% of exhaled particles were <5.0 µm in diameter. These findings regarding influenza virus RNA suggest that influenza virus may be contained in fine particles generated during tidal breathing, and add to the body of literature suggesting that fine particle aerosols may play a role in influenza transmission.

## Materials and Methods

### Location and recruitment

This study was conducted on a subset of subjects recruited in a randomized trial looking at the efficacy of face masks and hand hygiene to reduce influenza transmission in Hong Kong residents [41]. We recruited participants at three sites in Hong Kong, China during July through September of 2007. Subjects 12 years of age and older who presented with influenza-like illness (ILI) and were within the first 3 days of onset of symptoms were invited to participate in the study. A short questionnaire was used to record age, gender, clinical illness symptoms, medication use, medical and smoking history. Influenza-like-illness was defined as having a fever ≥37.8°C and two or more ILI symptoms (fever, cough, sore throat, runny nose, headache, malaise). Once written informed consent was obtained, nasal and throat swabs were collected and analyzed using a QuickVue Influenza A/B diagnostic test (Quidel Corp., San Diego, CA) to confirm influenza virus infection. A second set of nasal and throat swabs was collected and placed in viral transport media buffer (Earl's Balanced Salt Solution, 0.1% glucose, 0.5% bovine albumin and antibiotic) refrigerated at 2–8°C immediately after collection, stored at −20°C for up to 7 days, and then stored at −80°C until analyzed for laboratory confirmation of influenza virus infection by PCR. Only subjects who were determined to be infected with influenza A or B virus according to the rapid diagnostic test were asked to complete the exhaled breath collection. The institutional review boards of The University of Hong Kong and University of Massachusetts Lowell approved the research protocols. Subjects were compensated for their time and the inconvenience associated with participating in the study.

### Particle count and collection system

We collected exhaled breath from subjects using an Exhalair (Pulmatrix, Lexington, MA) a device which integrates optical particle counting technology (Airnet 310, Particle Measuring Systems, Boulder, CO) with airflow data obtained with a mass flow meter and also collects filter samples. Subjects breathed with a normal tidal pattern into an oro-nasal facemask (Hans Rudolph, Shawnee, KS) for approximately 20 minutes total. The face piece was connected to a respiratory T-valve which was equipped with a HEPA filter on the intake side to supply particle free, make-up air at very low resistance. A mass flow meter monitored inhalation and exhalation flows and the instrument computed and stored total and per breath flow and volume information. The outflow side of the T-valve was connected via tubing to first the optical particle counter and then to the filter sample collection part of the Exhalair device. The optical particle counter recorded particle counts in four size bins: 0.3 µm-<0.5, 0.5-<1 µm, 1-<5 µm and ≥5 µm. A vacuum pump pulled air through the tubing at 28.3 lpm into a real-time particle counting system during the exhaled breath particle characterization phase of the test (approximately 5 minutes). After completion of the particle counts, the outflow of the T-valve was attached to a 37-mm, 2-µm pore-size Teflon filter with a polymethylpentene (PMP) support ring (Pall Life Sciences, New York) and the vacuum pump was switched to pull through the filter during the particle collection phase of the test (15 minutes). Teflon filters were refrigerated at 2–8°C immediately after collection, stored at −20°C for up to 7 days, and then stored at −80°C until analyzed. T-valves and HEPA filters were disposed of after each use and masks were disinfected using 10% bleach and autoclaved prior to reuse.

### RNA extraction from exhaled breath samples

Influenza virus RNA collected from the exhaled breath on the Teflon filters was extracted using a Trizol-chloroform based method modified from a protocol developed for extraction of nasal swab and lavage samples [42]. Initially, the PMP support ring surrounding the Teflon filter was cut 6 to 8 times around the circumference of the filter and the filter was put into a 50-ml polypropylene centrifuge tube containing 400 µl of phosphate buffered saline (PBS). A 10 µl mixture containing 20 µg glycogen (Ambion, Austin, TX), 15 µg glycoblue (Ambion, Austin, TX), 50 ng of Human DNA (BD Biosciences, San Jose, CA) in PBS was added directly to each sample. Next, 750 µl of Trizol LS (Invitrogen, Carlsbad, CA) was added, the sample vortexed for 30 seconds and the liquid transferred to a 1.7 ml low protein binding tube (LoBind, Eppendorf, Westbury, NY). The tubes were placed on an MS2 Minshaker (IKA works, Guangzhou, China) for 10 minutes at 300 rpm. After mixing, 230 µl of chloroform (Sigma-Aldrich, St. Louis, MO) was added and samples were briefly vortexed and placed on the shaker at 300 rpm an additional 5 minutes. Samples were then centrifuged at 2100×g for 5 minutes and 700 µl of the aqueous supernatant was transferred to a tube containing 600 µl of 100% isopropanol (Sigma-Aldrich, St. Louis, MO) for RNA precipitation. After incubating for1 hour at room temperature, RNA was pelleted by centrifuging for 12 minutes at 20000×g at room temperature. The supernatant was decanted and 600 µl of 75% ethanol were added to wash the pellets. The RNA pellets were centrifuged for 5 minutes at 20000×g, the ethanol decanted and the pellets air dried for approximately 20 minutes. The RNA was suspended in 20 µl of nuclease-free water (Promega, Fitchburg, WI) and immediately converted to cDNA.

### RNA extraction from nasal swabs

RNA in the nasal and throat swabs was extracted from 140 µl of viral transport media using Qiagen QIAamp Viral RNA mini columns (Qiagen Corp., Valencia, CA) in accordance with the manufacturer instructions. The RNA was eluted in 100 µl of carrier buffer and immediately converted to cDNA. Unused RNA was stored at −80°C.

### Reverse transcription

cDNA was synthesized from purified and concentrated RNA using a High Capacity cDNA Reverse Transcription Kit (Applied Biosystems, Foster City, CA). A 20 µl total reaction volume was made with 10 µl RNA, 2 µl 10X RT buffer, 0.8 µl dNTP Mix (100 mM), 2.0 µl 10X RT random hexamer primers, 1.0 µl MultiScribe™ reverse transcriptase, 1 µl RNase inhibitor and 3.2 µl nuclease-free water. Synthesis was carried out in an ABI 9700 Thermocycler (Applied Biosystems, Foster City, CA) and reaction conditions were 25°C for 10 minutes, 37°C for 120 minutes, and 85°C for 5 seconds. cDNA samples were stored at −20°C.

### Quantitative PCR

Quantitative PCR was performed using an Applied Biosystems Prism 7500 detection system (Foster City, CA). Triplicate cDNA samples were analyzed in a 96-well plate with an adhesive film cover (Applied Biosystems, Foster City, CA). Each well contained 5 µl of cDNA template, 12.5 µl of 2X Taqman™ Universal PCR Master Mix (Applied Biosystems, Foster City, CA), 900 nM of each primer and 100 nM probe.

We tested three primer/probe sets on 17 nasal swab samples collected for this project in order to select the most sensitive for the exhaled breath and remaining nasal swab samples: a set used by the Centers for Disease Control, a set used at the Queen Mary Hospital Virology laboratory (QMH) and a set from the published literature [38]. Based on the sensitivity results from these tests the following set of primers and probe were selected to analyze the exhaled breath samples: for influenza A virus two forward primers 5′- GGA CTG CAG CGT AGA CGC TT-3′ and 5′- CAT CCT GTT GTA TAT GAG GCC CAT-3′, reverse primer 5′- CAT TCT GTT GTA TAT GAG GCC CAT- 3′, and probe 5′FAM- CTC AGT TAT TCT GCT GGT GCA CTT GCC A -3′TAMRA; for influenza B virus forward primer 5′- AAA TAC GGT GGA TTA AAT AAA AGC AA-3′, reverse primer 5′- CCA GCA ATA GCT CCG AAG AAA -3′, and probe 5′FAM CAC CCA TAT TGG GCA ATT TCC TAT GGC -3′TAMRA [38]. The primers and probe targeted the matrix (M) gene and the hemagglutinin (HA) gene for influenza A and B viruses respectively. Primers and probe were manufactured by Sigma-Proligo (Proligo Singapore Pty Ltd).

We constructed standard curves for the qPCR by making 1∶10 dilutions of cDNA made from QMH influenza A and B virus stocks. Influenza A/PR/8/34 and influenza B/Hong Kong/AE34/2002 virus stocks were grown at QMH on MDCK cells and purified via a sucrose density gradient. Once purified, 50 µl aliquots of each virus were extracted using the Trizol-chloroform method described previously, synthesized to cDNA using the High Capacity cDNA Reverse Transcription Kit (Applied Biosystems, Foster City, CA) and quantified by qPCR with a plasmid standard curve for each influenza virus. The virus concentrations calculated based on this method were 1.88×10^7^ and 1.66×10^6^ virus particles per µl for the influenza A and B virus stocks, respectively. The limit of quantification for the qPCR was 6 influenza A or B viral RNA particles per PCR well, with all three replicates crossing the qPCR fluorescence threshold within 37 cycles. For the exhaled breath filters and nasal swabs, samples were considered positive if at least one of the three replicates crossed the qPCR cycle threshold, but were only quantifiable if all three replicates crossed the cycle threshold. Because 25% of the cDNA was used per well and 50% of the extracted RNA was used to make cDNA, the total number of virus copies per filter was 8 times the average copy number per well.

### Influenza virus sub-typing

The RNA from the nasal swab samples positive for influenza A virus were shipped to the Centers for Disease Control Virus Surveillance and Diagnostics Branch Influenza Division. Samples were tested for H1 and H3 sub-types using quantitative PCR methods.

### Data analysis

Exhaled virus concentrations in exhaled breath were computed from the qPCR results and the Exhalair record of total exhaled volume during filter sample collection. Total particle concentrations and concentrations in each size bin were calculated for each breath by dividing the number of particles counted by the volume exhaled. We then averaged the particle concentrations over all breaths collected for each subject. Computations were performed in SAS for Windows (version 9.1.3, Cary, NC).
